# Individual supported work placements (ReISE) for improving sustained return to work in unemployed people with persistent pain: study protocol for a cohort randomised controlled trial with embedded economic and process evaluations

**DOI:** 10.1186/s13063-023-07211-5

**Published:** 2023-03-11

**Authors:** Pål André Amundsen, Martin Underwood, Kim Burton, Margreth Grotle, Ira Malmberg-Heimonen, Adnan Kisa, Milada Cvancarova Småstuen, Thor Einar Holmgard, Amy Martinsen, Jakob Lothe, Pernille Marie Stähr Irgens, Magnus Højen, Sølvi Spilde Monsen, Robert Froud

**Affiliations:** 1 School of Health Sciences, Kristiana University College, PB 1190, Sentrum, 0107 Oslo, Norway; 2 Warwick Clinical Trials Unit, Warwick Medical School, University of Warwick, Coventry, CV4 7AL UK; 3 Professor of Occupational Healthcare, University of Huddersfield, Queensgate, Huddersfield, HD1 3DH UK; 4 Department of Rehabilitation Science and Health Technology, Oslo Metropolitan University, St. Olavs Plass, P.O. Box 4, 0130 Oslo, Norway; 5 Department of Social Work, Child Welfare and Social Policy, Oslo Metropolitan University, St. Olavs Plass, P.O. Box 4, 0130 Oslo, Norway; 6 Department of Nursing and Health Promotion, Faculty of Health Science, Oslo Metropolitan University, St. Olavs Plass, P.O. Box 4, 0130 Oslo, Norway; 7 User representative from the Norwegian Back Pain Association, Fjellhagen, P.O. Box 9612, 3065 Drammen, Norway; 8 Department of Research, Innovation and Education, Division of Clinical Neuroscience, Research and Communication Unit for Musculoskeletal Health (FORMI), Oslo University Hospital, Ullevål, Building 37B, P.O. Box 4956 Nydalen, 0424 Oslo, Norway; 9 Co/FORMI, The Norwegian Council for Musculoskeletal Health, Oslo Universitetssykehus, Nydalen, P.O. Box 4956, 0424 Oslo, Norway; 10 Manpower, Lakkegata 53, 0187 Oslo, Norway

**Keywords:** Persistent pain, Quality of life, Supported employment, Vocational rehabilitation, Unemployment, Work disability, Return to work, Labour market participation, Cohort randomised approach, Case management

## Abstract

**Background:**

Around one-third of workdays lost in Norway are due to musculoskeletal conditions, with persistent (chronic) pain being the most frequent cause of sick leave and work disability. Increasing work participation for people with persistent pain improves their health, quality of life, and well-being and reduces poverty; however, it is not clear how to best help unemployed people who have persistent pain to return to work. The aim of this study is to examine if a matched work placement intervention featuring case manager support and work-focused healthcare improves return to work rates and quality of life for unemployed people in Norway with persistent pain who want to work.

**Methods:**

We will use a cohort randomised controlled approach to test the effectiveness and cost-effectiveness of a matched work placement intervention featuring case manager support and work-focused healthcare compared to those receiving usual care in the cohort alone. We will recruit people aged 18–64, who have been out of work for at least 1 month, had pain for more than 3 months, and want to work. Initially, all (*n* = 228) will be recruited to an observational cohort study on the impact of being unemployed with persistent pain. We will then randomly select one in three to be offered the intervention. The primary outcome of sustained return to work will be measured using registry and self-reported data, while secondary outcomes include self-reported levels of health-related quality of life and physical and mental health. Outcomes will be measured at baseline and 3, 6, and 12 months post-randomisation. We will run a process evaluation parallel to the intervention exploring implementation, continuity of the intervention, reasons for participating, declining participation, and mechanisms behind cases of sustained return to work. An economic evaluation of the trial process will also be conducted.

**Discussion:**

The ReISE intervention is designed to increase work participation for people with persistent pain. The intervention has the potential to improve work ability by collaboratively navigating obstacles to working. If successful, the intervention may be a viable option for helping people in this population.

**Trial registration:**

ISRCTN Registry 85,437,524 Registered on 30 March 2022.

**Supplementary Information:**

The online version contains supplementary material available at 10.1186/s13063-023-07211-5.

## Administrative information

**Table:** 

Title {1}	Study protocol for a cohort randomised controlled approach to study the effectiveness and cost-effectiveness of individual supported work placements (ReISE) for improving sustained return to work in unemployed people with persistent pain
Trial registration {2a and 2b}	The trial is registered in ISRCTN (Ref. no. 85437524. Prospectively registered 30/03/2022) https://www.isrctn.com/ISRCTN85437524
Protocol version {3}	31.01.2023, v1.0
Funding {4}	Norwegian Research Council; Collaborative Project to meet Societal and Industry-related Challenges scheme. Grant number 326732/ABHO The funder played no part in the study design and will play no part in the collection, management, analysis, and interpretation of the data; writing of the report; or the decision to submit the report for publication
Author details {5a}	^1^School of Health Sciences, Kristiana University College, PB 1190, Sentrum, 0107, Oslo, Norway ^2^Professor of Occupational Healthcare, University of Huddersfield, UK, Queensgate Huddersfield, HD1 3DH ^3^Department of Social Work, Child Welfare and Social Policy, Oslo Metropolitan University, St. Olavs Plass, P.O. Box 4, 0130, Oslo, Norway ^4^Department of Rehabilitation Science and Health Technology, Oslo Metropolitan University, St. Olavs Plass, P.O. Box 4, 0130, Oslo, Norway ^5^Warwick Clinical Trials Unit, Warwick Medical School, University of Warwick, Coventry, CV4 7AL, UK ^6^Department of Nursing and Health Promotion, Faculty of Health Science, Oslo Metropolitan University, St. Olavs Plass, P.O. Box 4, 0130, Oslo, Norway ^7^User representative from the Norwegian Back Pain Association, P.O. Box 9612 Fjellhagen, 3065, Drammen, Norway ^8^Department of Research, Innovation and Education, Division of Clinical Neuroscience, Research and Communication Unit for Musculoskeletal Health (FORMI), Oslo University Hospital, Oslo, Norway, Ullevål, Building 37B, P.O. Box 4956 Nydalen, 0424 Oslo ^9^The Norwegian Council for Musculoskeletal Health, Co/FORMI, Oslo Universitetssykehus, P.O. Box 4956 Nydalen, 0424 Oslo, Norway ^10^Manpower Lakkegata 53, 0187 Oslo, Norway
Name and contact information for the trial sponsor {5b}	Høyskolen Kristiania, Ernst G Mortensens Stiftelse Postboks 1190 Sentrum, 0107 Oslo Enquiries to Our ref.: Professor Hans-Christian Åsheim, Dean, School of Health Sciences Telephone: + 47 454 35 209 Email: hans-christian.asheim@kristiania.no
Role of sponsor {5c}	The sponsor played no part in the study design and will play no part in the collection, management, analysis, and interpretation of the data; writing of the report; and the decision to submit the report for publication The sponsor has the overall responsibility for effective arrangements to be in place to set up and run the trial and provide regulatory advice as well as act as the legal signatory

## Introduction

### Background and rationale {6a}

Persistent (chronic) pain is the most frequent cause of sick leave and work disability in Norway; accounting for 11% and 9% of employed workers, respectively [1]. Most chronic pain is of musculoskeletal origin [2]. In 2019, around one-third of work days lost in Norway were due to musculoskeletal conditions [3]. Increasing work participation in sick and disabled populations improves health outcomes, reverses adverse effects of worklessness, reduces poverty, and improves quality of life and well-being [4]. In 2016, the costs of musculoskeletal conditions to the Norwegian health service were NOK 18 billion, and the total socio-economic costs were estimated to be NOK 255 billion [5]. Disability benefits increased between 2011 and 2020, and the percentage of people outside the labour market increased between 2018 and 2020 (in both cases by around 1.5%) [6, 7]. The average age of people reporting persistent pain in Norway is 48 (SD = 16) [8]. However, these people may have many remaining working years to contribute [9].

It is not clear how to best help unemployed people with persistent pain return to work (RTW). In Norway, usual care for people with persistent pain is interdisciplinary, featuring pain management services supported by physicians, psychologists, physiotherapists, and nurses [10]. Employment services in Norway take an individualised approach, often including assessments of work ability, training, and vocational rehabilitation programmes involving traineeships in sheltered businesses [11]. However, these services are not specifically tailored for people with pain. Effective vocational rehabilitation requires a combination of work-focused healthcare and supportive workplaces; unfortunately, the two are generally not coordinated [12]. Without intervention, return to work is unlikely after 2 years of unemployment [13]. Return to work rates for traditional vocational rehabilitation in the unemployed (rather than sick-listed) pain population are unclear. In 2020, we described the results of a feasibility study, done in the English Midlands, of an adapted supported work placement intervention, which featured work placement provision with case management support [13]. In this study, case managers were trained to act as a bridge between systems and institutions (e.g. between health care providers and the workplace), engage people about obstacles to work, and collaboratively agree on a return-to-work plan. We also provided supportive work placements. The developed intervention was acceptable and valued by the people involved, with around 20% of the sample obtaining paid employment within the short 6-month follow-up. The study suggested that self-perception of satisfactory functioning in role as well as management of identified obstacles may be key to increasing confidence for this group. As the study focused on feasibility, the effectiveness and cost-effectiveness of the intervention are still unknown.

### Objectives {7}

The primary objective of this study is to establish whether a supported work placement intervention with biopsychosocial case management is more effective and cost-effective than usual care for improving sustained return to work in unemployed people with persistent pain who want to work.

The secondary objectives include the following:

- To investigate the impact of being unemployed with persistent pain on health-related quality of life, including physical function, anxiety, depression, fatigue, sleep disturbance, social function and activities, pain interference, bothersomeness, pain intensity, mental well-being, and confidence in return to work and use of social care resources
- To define usual care and explore what type of existing services this population typically receive
- To investigate the influence of demographics on enrolling on the cohort study
- To explore the mechanisms of action in people who achieve sustained return to work and identify possible predictive factors of sustained return to work
- To describe the delivered processes in terms of fidelity, context, reach, dose, and adherence and the implementation of the intervention.

### Trial design {8}

**Fig. 1:**
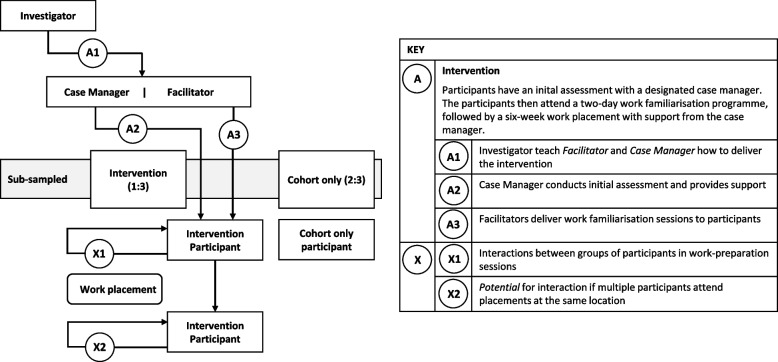
Cascade diagram showing the intervention delivery

**Fig. 2:**
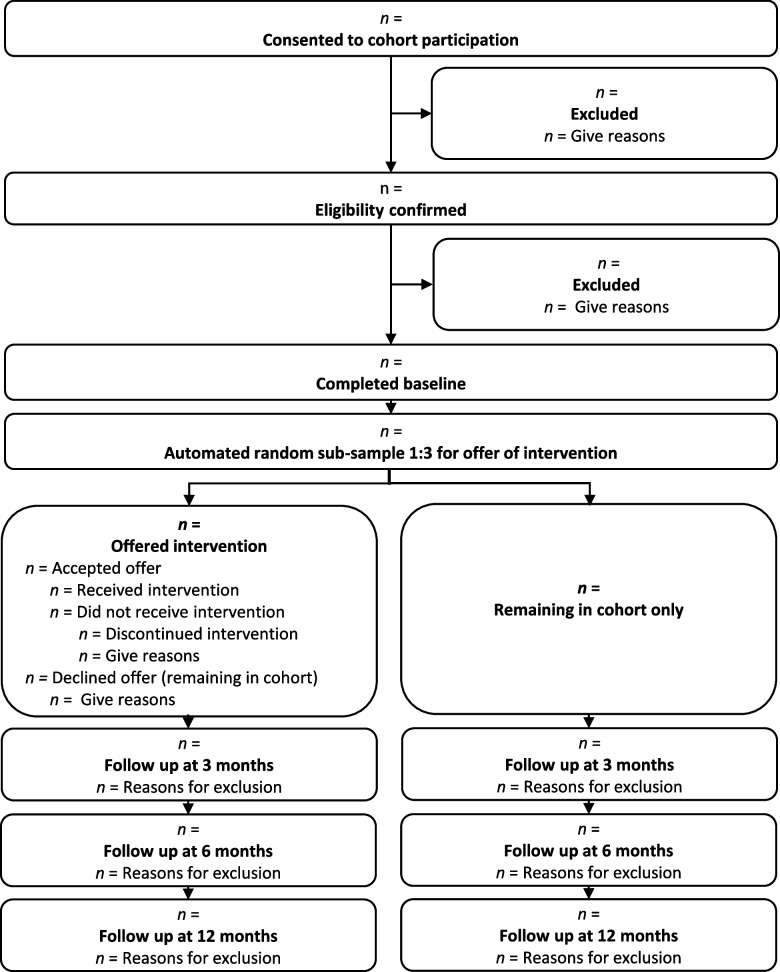
CONSORT guideline extension for reporting randomised controlled trials conducted using cohorts and routinely collected data

We will use a single-site cohort randomised approach. The trial will be conducted within a superiority framework. Initially, all eligible and consenting participants will be recruited for an observational cohort study. After baseline measurement, we will randomly select one in three, to whom we will offer the intervention and request consent for intervention participation. Figure 1 illustrates the plan for the delivery of the intervention using a cascade diagram [14]. Assessments will be made at baseline and at 3-, 6-, and 12-month follow-up, and the primary outcome of return to work will be obtained from questionnaire and registry data at these time points. We will also use registry data at all follow-up time points to estimate hospital and community health and social care use for our economic analysis from a personal social services perspective. A pre-pilot informs logistical processes and pre-pilot participants may be included in the main trial if there are no substantial protocol amendments during the pre-pilot, or as a result of piloting (i.e. no alteration in the cohort and intervention delivery). Parallel to the intervention, we will run a process evaluation exploring implementation, continuity of the provided package, reasons for participating and declining participation, and mechanisms behind cases of sustained return to work. The study will be reported following the CONSORT guideline (Consolidated Standards of Reporting Trials; http://www.consort-statement.org/) extension for the reporting of randomised controlled trials conducted using cohorts and routinely collected data (Fig. 2) [15]. The study protocol has been written using the structured protocol template for Standard Protocol Items: Recommendations for Interventional Trials (SPIRIT) Checklist for intervention trials [16].

## Methods: participants, intervention, and outcomes

### Study setting {9}

Following the exploration of remote case management support and work familiarisation by video-link in the pre-pilot, we aim to recruit nationally for the main trial. However, we anticipate most recruitment will be in the Oslo area. Placements will be sought in diverse areas including administration, warehouse, transport, media and marketing, IT, sales and service, finance, accounting, and payroll, in line with participant preference and skill.

### Eligibility criteria {10}

We will include people aged 18–64 who have been unemployed for at least 1 month, have had pain for more than 3 months, and who want to work. We will exclude those aged 65 or over as their future time in work is likely to be limited. We will exclude people who do not have sufficient Norwegian or English language skills to give consent. This criterion is in line with the fluency required to enter the open labour market in Norway.

### Who will take informed consent? {26a}

We will use online and offline recruitment channels (see SPIRIT item 15 for further details). Participants will be given a participant information leaflet describing essential information about the study. Potential participants will reply to an ‘expression of interest’ form, including self-reporting of eligibility. Additionally, contact information for the research team will be provided within the participant information leaflet and expression of interest, encouraging to make contact if they have questions. Based on preferences, the consent form will be delivered by post or through Nettskjema (UiO, Oslo) for those engaging online. Before providing consent, potential participants must confirm that they have read and understood the participant information leaflet. After receiving the consent form, a trial working group member will phone participants to confirm eligibility before inviting the participant to complete a baseline questionnaire. After completing the baseline questionnaire, one-in-three people will be randomly selected to be offered the intervention. The initial cohort study consent form will request consent to be approached for the intervention. A member from the trial working group will contact those selected by phone, providing information and to ask if they have any questions and if they might like to participate. An email or post package, based on their previous preferences, will be sent to those interested after the call, and contain a participant information leaflet describing the intervention and a consent form. If there is no response to the phone call, the email or post package will note that a member from the trial working group will contact by phone to ask if the participant has any questions or requires further information. The participant information materials and informed consent form are available from the corresponding author on request.

### Additional consent provisions for collection and use of participant data and biological specimens {26b}

The study does not collect biological data or specimens.

### Interventions

#### Explanation for the choice of comparators {6b}

In line with the pragmatic trial design, the study protocol will not restrict access to usual care [17]. Usual care for people with persistent pain is interdisciplinary in Norway, featuring pain management services supported by physicians, psychologists, physiotherapists, and nurses [10]. In general, employment services take an individualised approach, including work ability assessments, training, and vocational rehabilitation programmes involving traineeships in sheltered businesses [11]. A secondary objective of the trial is to explore what existing employment services this specific population typically receives as usual care.

#### Intervention description {11a}

First, we will train two to four people with prior experience of working with pain patients (e.g. nursing, manual/occupational therapy, or psychology) to function as case managers for participants and identify obstacles to return to work using the ‘Psychosocial Flags Framework’ and a set of stem questions [13, 18]. Using a biopsychosocial approach, the case managers will identify and create a plan to tackle obstacles across personal, workplace, and social domains, documenting these in a return-to-work plan. Case managers will receive training on how to do this using a toolkit used as the evidence base for case management and for conducting work familiarisation sessions. Case managers will also receive training on pain management strategies and the Norwegian benefits system, as they will be unaffiliated with the Norwegian Labour and Welfare Administration (NAV), which our user input suggests may be important.

The trained case manager will contact participants by phone to make an initial assessment, explore work aspirations and skills, and help the participant to identify work obstacles. A face-to-face or video will be offered if the case manager considers one needed. Through collaboration with Manpower, a large recruitment company in Norway, the case manager will match the participant with a placement, based on skills, aspirations, and suitability. From their network of associates, Manpower will provide 6-week part-time work placements of up to 16 h per week (as participants receiving work assessment allowance (*Arbeidsavklaringspenger*) may have limits on how much they are allowed to work). Once a placement is arranged, the case manager, the participant, and the placement manager will meet to agree on a return-to-work plan and discuss whether any work accommodations are needed to overcome identified obstacles to returning to work [19, 20]. While Manpower aims to provide all of our placements, we may approach additional placement providers if needed.

Our support model features a work-familiarisation session to help embed the person into their role and discuss work-focused pain management. The session will be run in small groups over 2 days by one or two facilitators in required geographic locations and by video link when outside of Oslo. The case manager will then provide regular telephone or video support to the participant and placement manager, which is known to be effective [21]. If needed, the case manager may suggest referrals to existing health services, such as cognitive behavioural therapy and physiotherapy [12]. The case manager will also aid clinicians in providing work-focused healthcare by providing existing evidence-based resources. Similar materials are given to all participants and placement providers and include culturally translated versions of (1) *advising patients about work*, (2) *health and work*, and (3) *work and health* originally commissioned to support the UK government work ability initiatives [22–24]. These will be available in Norwegian and English, following the success of offering these languages in a trial of motivational interviewing that is being conducted for people sick-listed with pain in Norway [25]. A paper on the cultural adaptation of these materials will be reported elsewhere.

#### Criteria for discontinuing or modifying allocated interventions {11b}

Participants will be allowed to withdraw their consent to participate in the cohort or the intervention at any time without personal disadvantages or having to give a reason. If a participant withdraws from the cohort or intervention, no additional data will be collected, but any data collected up to that point will be included in the analysis as permitted by General Data Protection Regulation (GDPR) when using public task as the legal basis (GDPR Art. 6(1)e, 9(2)j) [26]. Information about voluntary participation and the possibility to withdraw at any time will be emphasised in the participant information leaflet, while information on data processing will be provided in the privacy notice. There will be no special criteria for discontinuing or modifying allocated interventions; other than required adjustments in case of COVID-19 restrictions; placement provision will adhere to any adaptive working arrangements as may be required, while contact between participant; and case manager and/or within work familiarisation sessions will in this case be completed by phone and video.

#### Strategies to improve adherence to interventions {11c}

##### Participant adherence

To increase adherence to data collection and decrease the risk of dropout, participants will be given reminders, and their response rates will be monitored by the trial manager. The trial manager will be responsible for reminding participants of data collection appointments and being available to answer questions and concerns. Participants in the intervention group will be matched with a case manager who provides support throughout the 6-week placement and will answer any questions the participant may have about the intervention.

##### Implementation and staff adherence

Case managers will use a standardised form developed for reporting contact with participants, and templates for their initial assessment and return to work plan ensuring that case managers adhere to the same procedures. The case manager and facilitator training component, resource package, and standardised templates are informed from experience from the UK feasibility study and modified for Norwegian consumption [13]. The training will be recorded so each staff member can revisit the training component as needed. Additionally, members of the trial working group will participate in the training, thus being able to provide a stand-in service if necessary. Other strategies to improve adherence include trial manager liaison with case managers, staffing provider, and placement manager.

### Adherence monitoring

Monthly meetings with the Trial Management Group will include a review of monitoring and oversight, deviations, violations, breaches, and risk assessment. Spirit items 5d and 21a describe the composition and role of the Trial Steering Committee (TSC) and Data Monitoring Committee (DMC), which both are important for adherence monitoring. The process evaluation will include monitoring of the numbers and reasons for participant drop-out and procedures used to facilitate continued participation, fidelity to the conceptual model and the extent to which the familiarisation sessions and intervention were implemented as designed; reasons for health referrals, and intervention acceptability and adherence [27].

### Relevant concomitant care permitted or prohibited during the trial {11d}

Participants in both the cohort group and the intervention group will be free to seek other forms of care or use any medication during the trial. Within the intervention, the case manager will support any participant who needs to consult for work-related healthcare, which will be registered as part of secondary outcomes in the study.

### Provisions for post-trial care {30}

The intervention aims to tackle obstacles to work and create an individual plan to return to work. Some participants may be referred to work-related healthcare by their general practitioner during the intervention. In cases of prolonged need for healthcare, the individuals will maintain a dialogue with their general practitioner, this being independent of the intervention.

### Outcomes {12}

### Primary outcome measure

#### Sustained return to work

There is no consensus in the literature about what constitutes a sustained return to work. In trials of return-to-work interventions, outcome measures have been expressed with employment success constituting as little as one day of paid work [28]. However, we consider the focus on sustained return to work more useful and relevant [29]. Thus, in this study, the primary outcome will be the proportion of participants achieving a sustained return to work, which we define as the first 4 weeks of 50 to 100% work. We will examine the differences between the cohort group and intervention group, with differences at 12 months being the primary end-point. For a sustained return to work, the primary outcome data will be collected using Norwegian Labour and Welfare Administration registry data, and self-report data at 3, 6, and 12 months from baseline measurement in the case of missing registry data.

### Secondary outcome measures

Secondary outcome measures will be health-related quality of life, work ability, pain and pain intensity, and satisfaction with care, and we will examine the between-group differences at 3, 6, and 12 months after baseline measurement. These outcomes are selected to reflect important domains associated with work, unemployment, and return to work [30–33]. The EQ-5D-5L will be used to measure the potential changes in participants’ self-reported health-related quality of life within five domains: mobility, self-care, usual activities, pain/discomfort, and anxiety/depression. The WEMWBS measures mental well-being [34], while the PROMIS-29 will be used as the outcome for related domains: anxiety, depression, fatigue, pain (intensity and interference), physical function, sleep disturbance, and social participation [35]. Work-related outcomes include numeric scale measures in confidence in return to work and work ability a return to work self-evaluation and offers of employment. For those who have returned to work, measures include usual work patterns, job satisfaction, and the WRQF, an outcome measure linking a person’s health to the ability to meet work demands [36].

### Cost-effectiveness

We will use registry data: NPR (*Norsk Patientregister*), KPR (*Kommunalt pasient- og brukerregister*), and KUHR (*Kontroll og utbetaling av helserefusjoner*), to estimate hospital and community health care and social care resource use from three months prior to baseline measurement and 3, 6, and 12 months after baseline measurement. To support the economic evaluation, our own questionnaires include items of health and social care use in a broader sense than collected registry data. All participants in the study will be assessed with self-reported measures and registry data comprising work-related data and health and social care use.

At baseline, we will collect demographic data and details of health care use in the past 3 months as well as health-related outcomes using the following validated questionnaires, which will be offered in Norwegian or English as preferred: health-related quality of life (EQ-5D-5L) [37, 38], the Warwick-Edinburgh Mental Well-being Scale [34, 39], PROMIS-29 [40, 41], and the Work Ability Score (as a single item taken from the Work Ability Index) [42, 43]. The EQ-5D-5L has been translated into Norwegian using the EuroQul Group translation methodology and Solberg et al. concluded that the instrument is reliable [44, 45]. Garratt et al. compared the EQ-5D-5L to the EQ-5D-3L, which has undergone limited evaluation in Norwegian patients with LBP, to examine floor and ceiling effects, response consistency, and concurrent validity, and found the 5L preferable to the 3L [46]. Garratt et al. have also reported Norwegian population norms for the instrument [47]. Work to determine a Norwegian value set is ongoing and anticipated to be available by the end of the trial [37]. Smith et al. did a confirmatory factor analysis and examined criterion validity and measurement invariance of their Norwegian version of the WEMWBS, reporting that it appears to measure mental well-being well and commending it for use [48]. Garratt et al. reported the Norwegian version of the PROMIS-29 to be a reliable and valid generic self-reported measure of health in the Norwegian general population, having done confirmatory factor analysis to assess structural validity and also examining concurrent validity [49]. The Work Ability Score item in Norwegian to our knowledge has not undergone any formal validation work, which we will acknowledge as a limitation. However, normative data from the Norwegian general population for the question is available for comparison [50].

We will collect follow-up data at 3, 6, and 12 months from baseline measurement, where we additionally will ask about return to work, job offers, or usual care and services, and then, for those who have returned to work: satisfaction with work, the Work Role Functioning Questionnaire (WRFQ) [51, 52], and return to work self-efficacy [53–55] whether work makes the pain worse, healthcare and social care use, medication, and the amount of time they have been at work (Table 1). The WRQF has been cross-culturally adapted into Norwegian using guidelines proposed by Beaton et al. [56, 57]. Johansen et al. examined the internal consistency reporting Cronbach’s alpha coefficients for sub-scales were well-zoned, ranging between 0.80 and 0.90 [57]. The return-to-work self-efficacy scale has been translated into Norwegian and been assessed using principal components analysis, in addition to studies of internal consistency and concurrent validity. Cronbach’s alpha was high enough to indicate the potential for redundant items, although PCA outlined a three-factor structure matching the original instrument. The authors conclude that the instrument has satisfactory cross-cultural validity [55].

In general, there is an absence of work examining reliability and responsiveness of these instruments, which we will acknowledge as a limitation.

**Table 1:** Outcome domains, measurement instruments, and measurement time points

Domain	Measurement instrument or key items within the questionnaire package	Abbreviation	T0	T1	T2	T3
Demographic	N/A		X			
Pain	Troublesome grid		X			
	Pain duration		X			
Work-related baseline	Educational level		X			
	Unemployed duration		X			
	Looking for work duration		X			
	Reasons for wanting to RTW		X			
Health-related quality of life	Health-related quality of life	EQ-5D-5L	X	X	X	X
	Warwick-Edinburgh Mental Well-being Scale	WEMWBS	X	X	X	X
	Patient Reported Outcome Measurement Information System 29	PROMIS 29	X	X	X	X
	Pain intensity		X	X	X	X
Details of care received and services used	Registry data: Norwegian patient register, Municipal patient and user register, and Control and payment of health reimbursements		X	X	X	X
	Received any care or help for pain last three months* or since the last questionnaire		X*	X	X	X
	List of used medications in last three months* or since the last questionnaire		X*	X	X	X
Work-related	Dimensions of Work Ability		X	X	X	X
	Confidence in RTW		X	X	X	X
	RTW self-evaluation		X	X	X	X
	Job offers			X	X	X
	Return to work			X	X	X
For those who return to work	Satisfaction with work			X	X	X
	Pain interference with work			X	X	X
	Work pattern			X	X	X
	Work-role functioning	WRFQ		X	X	X
Sustained RTW (Primary)	Registry data from the Norwegian Labor and Welfare Administration			X	X	X
Adverse events	Case Report Forms			X	X	X

*Last three months for baseline measurement

### Participant timeline {13}

**Fig. 3:**
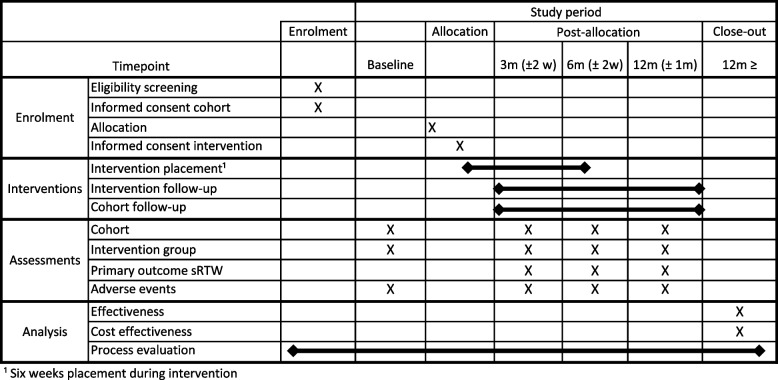
Participant timeline

A participant timeline was created according to the SPIRIT guidance and is presented in Fig. 3 [58]. Following admission to the cohort study, baseline measurement will be completed. After baseline measurement, one in three will be randomly selected to be invited to the intervention (see SPIRIT item 16a). If wishing to join the intervention, the participant will be assigned a case manager who will conduct the initial assessment. The case manager will then match the participant to a suitable work placement in collaboration with Manpower. The participant, case manager, and placement supervisor will agree and sign a return to work plan, and the participant will attend a 2-day work familiarisation session prior to the 6-week placement. Self-reported follow-up questionnaires will be completed by all participants 3, 6, and 12 months after baseline.

### Sample size {14}

The sample size calculation is based on detecting a difference of 20% between the intervention group and the remaining cohort achieving a sustained return to work (1:3 allocation ratio). Assuming a cohort return to work rate as high as 10%, with a power of 80% and a 5% significance level, we will need data on 176 participants (132 control and 44 intervention) [13]. Allowing for a 20% loss to follow-up and a crude 14% inflation in the intervention group for an estimated design effect due to clustering by the case manager (assumptions here are an ICC of 0.01, smallest cluster size of 7 and largest of 16, and so a design effect of 1.14), we will aim to recruit 228 people [59]. If a 1:3 ratio allocation provides insufficient throughput for us to regularly form small groups of people for the work preparation session due to the proportion of people not accepting the intervention being high, we may switch to target overall balanced randomisation (1:2). In this case, our overall required sample size will be reduced to 190 in total, with the above inflations (with the same assumptions the estimated design effect will be 1.11 in this case) [59].

### Recruitment {15}

Over an anticipated recruitment period of 1 year, we will mount an advertising campaign across social media, pain groups, and charities (including pain and rheumatological charities: *Ryggforeningen*, *Norsk Revmatikerforbund*, *Foreningen for Kroniske smerter*), public facilities, and *Frisklivssentralene* (i.e. ‘healthy living’ centres, which provide help with health and work matters). Manpower will aid recruitment using its online channels. We will offer NOK 200 of high street vouchers to those joining the cohort, which we have previously found to help recruitment [60, 61].

### Assignment of interventions: allocation

#### Sequence generation {16a}

Randomisation will feature minimisation and be automated using a mix of Stata scripting (StataCorp, College Station, TX) and bash programming to keep personal data encrypted. The research team developed the minimisation script which at its core features the Stata program rct_minim [62, 63]. The first participant is randomly allocated with equal probability, and then for each subsequent participant, there is a two-thirds probability that allocation will be to the group that best minimises the imbalance on the selected factors (i.e. gender on three levels (male, female, or prefer not to say) and three levels of age (18 to 29, 30 to 49, and 50 and over)) [64–66].

#### Concealment mechanism {16b}

No one in the study team will be able to predict or influence who will receive an offer to join the intervention or know the allocation sequence in advance, as this is run iteratively after each baseline completion.

#### Implementation {16c}

The trial manager will run the script at least twice a week, and in cases where cohort participants are allocated to the intervention, the trial manager will retrieve contact information and give an invitation to participate in the intervention (see SPIRIT item 26a).

### Assignment of interventions: blinding

#### Who will be blinded {17a}

Participants receiving the intervention, study staff directly involved in the trial, and placement providers cannot be blinded due to the nature of the study. It will not be possible to blind the trial statistician to allocation status, due to the nature of sub-sampling 1:3 to be offered the intervention. In the case we switch to 1:2 randomisation, and groups are balanced, the statistician will be blinded.

#### Procedure for unblinding if needed {17b}

Not applicable as participants and study staff cannot be blinded.

### Data collection and management

#### Plans for assessment and collection of outcomes {18a}

The planned time points and methods for outcome data collection are summarised in Table 1, with detailed information regarding primary and secondary outcome measures provided in SPIRIT item 12. Participants will be offered to complete self-reported measures in English or Norwegian (based on the preferred language as stated when providing informed consent) and on paper (by postage) or in a digital format (by email) using Nettskjema. If the measures are completed using Nettskjema, we will receive complete data as questionnaire items are mandatory. Measures conducted on paper will be entered independently by two research team members. For our primary outcome of sustained return to work, we plan to use registry data from NAV, although we could use our own questionnaire data, in the case of missing registry data. For details of care received and services used, we will use registry data. The process evaluation will utilise a mix of data (see SPIRIT items 12 and 19) collected from the questionnaires, expression of interest forms (with consent), and registry data in addition to field notes, qualitative interviews with participants, case managers, placement managers, and diary records.

#### Plans to promote participant retention and complete follow-up {18b}

We have several strategies to promote participant retention, including strategies noted in SPIRIT item 11c. Case managers are important to promote retention as they will give support and aid participants in the intervention. We will give participants the option to receive their questionnaires either on paper or digitally. High street vouchers will be provided for all participants (both those in only the cohort and those offered the intervention), and travel expenses to work placements will be reimbursed for the intervention group [67]. Calls and reminder letters/emails will be used to encourage data return at all time points [68, 69].

### Data management {19}

The project will generate a mix of primary electronic and paper data, which will be retained for a minimum period of 10 years after study completion. Case report forms will be developed to collect all required study data. All electronic data will be collected using Nettskjema, which is a data collection system that ties to TSD (Service for Sensitive Data, UiO Oslo) using AES-256 encryption with public key technology, on which the Stata instance will run our script to manage and randomise. Paper-based forms, case report forms and the trial master file will be stored (deidentified) at Kristiania University College (KUC) in a locked filing cabinet and a restricted access room for a minimum period of 10 years. Questionnaires retrieved by post will manually be entered in a dummy Nettskjema form to ensure all data are gathered and securely stored in TSD. Our script merges each response from Nettskjema (expression of interest, consent, baseline, and follow-up) into a set of encrypted files containing contact information, consent responses and other directly identifiable data, and a pseudonymised data file merging baseline and follow-up responses, removing and transferring identifiable data before encrypting these as separate files. Data stored at TSD will be accessible by the trial working group and the trial statistician at Oslo Metropolitan University (OsloMet). The statistician at OsloMet will use the Stata software provided by TSD so that data never need to leave the TSD environment. Registry data will be retrieved as encrypted files directly into TSD from the given registry. Data collected by qualitative methods (focus group and individual interviews) will first be transcribed, then stored digitally (anonymised) in TSD for analysis using the NVIVO software (also via TSD infrastructure). We will explore using TSD’s dictaphone app (‘Nettskjema-diktafon’) which encrypts the file on the phone/tablet and sends the file to TSD for transcription and analysis. If the dictaphone app is sufficiently accurate, we will use this software; if not, we will use professional transcription services, anonymising transcripts. In this case, digital recordings of audio interviews will be securely erased immediately following approved transcription and electronic copies of transcripts will be stored in the TSD. Data Processing Agreements exist between KUC and OsloMet, and KUC and Manpower. Personally identifiable data will need to be shared between KUC and Manpower (e.g. to facilitate matching placements by providing details of the participants to be placed). Data shared between KUC and OsloMet will be pseudonymised. Sharing of data between KUC, Manpower, and case managers will be done using asymmetric encryption, where each team has a corresponding private decryption key. Data will be owned by KUC.

### Confidentiality {27}

We will take several steps to maintain confidentiality. All study staff and investigators will protect the rights of the trial’s participants to privacy and informed consent. All information collected will be handled strictly in accordance with the consent provided, always adhering to the GDPR and to Good Clinical Practice standards. The study has been approved by the Norwegian Regional Committee for Medical and Health Research Ethics (Reference no. 402918), and a Data Protection Impact Assessment has been conducted to safeguard privacy in collaboration with the Norwegian Centre for Research Data. The data management plan noted in SPIRIT item 19 will ensure confidentiality in the data collected.

### Plans for collection, laboratory evaluation, and storage of biological specimens for genetic or molecular analysis in this trial/future use {33}

This trial does not involve the collection, laboratory evaluation, and storage of biological specimens.

### Statistical methods

#### Statistical methods for primary and secondary outcomes {20a}

A detailed statistical analysis plan has been finalised and agreed prior to analysis by the research team. We will report all participant flow in line with the CONSORT guideline extension for reporting RCTs conducted using cohorts and routinely collected data showing withdrawal and loss to follow-up [15]. Analyses will be conducted within TSD using Stata v17 or above (Stata Corporation, College Station, TX). Participant characteristics and outcomes will be summarised as mean and standard deviation for normally distributed continuous data or with median and ranges for data with skewed distributions or counts and percentage for categorical data. The primary analysis approach will be ‘intention-to-treat’. To account for the trial design featuring clustering in the intervention arm, generalised linear (GLM) mixed effects models will be used to estimate the between-group differences for both primary and secondary continuous outcomes, adjusting for age, gender, and duration of pain, with a random intercept for case manager. For example, we will analyse the between-group differences in sustained return to work (a binary outcome) using a generalised mixed-effect model with logit link function, the above covariates, and fitting a random effect in case manager allocation.

Secondary outcomes will also be analysed using generalised mixed models, using link functions appropriate to the outcome (e.g. where outcomes are continuous, we will use a canonical link function). In all cases, we will adjust for the same covariates (i.e. age, gender, and duration of pain). The adjusted treatment effect estimates (mean difference) between the groups will be presented as point estimates within a 95% confidence interval. Our outcome measures are largely independent and no correction for multiple testing will be done. *P*-values < 0.05 will be considered statistically significant. All tests will be two-sided.

An economic evaluation will be conducted from the personal social services perspective [70]. At 3, 6, and 12 months post-randomisation, we will also use registry data to estimate hospital and community health and social care resource use. Current Norwegian unit costs will be applied to value total resource use in both groups. Responses to the EQ-5D-5L and SF-12 will be converted into multi-attribute Norwegian utility scores [71, 72]. Results will be presented using incremental cost-effectiveness ratios and cost-effectiveness acceptability curves. This accommodates sampling uncertainty and varying levels of willingness to pay for an additional quality-adjusted life year. We will consider a decision-analytical model to determine the expected costs and consequences of the intervention arm and care as the usual arm.

The process evaluation will use both quantitative and qualitative analysis. To investigate the mechanisms of sustained return to work, we will collect qualitative data from two to three focus groups with participants, individual interviews with case managers and placement managers, field notes, and observation techniques. The analysis of qualitative data will be thematic, and we will use the framework method in which data obtained during the intervention will be used to help build the thematic framework, using the software package NVivo (QSR International, Victoria) [73]. Quantitative data from the trial will be used to model predictors of joining the cohort and intervention, using anonymised NAV routine data (e.g. demographics, length of unemployment, and type of employment) and data from questionnaires (e.g. health-related quality of life and dimensions of work ability). Potential predictors will be selected through a trial managing group brainstorming session following a review of the available variables. Potential predictor variables will first be correlated in matrices and any highly colinear variables removed. We will then fit crude logit models, in which joining the intervention will be the outcome. Significant variables will subsequently be entered into adjusted logit models with a view to building a model with respect to both model parsimony and variance explanation.

### Framework

A superiority hypothesis testing framework will be used to compare the outcomes in the intervention group to the cohort-only group.

### Interim analyses {21b}

An independent data monitoring committee (DMC) will be convened to review material in relation to these objectives. The trial statistician will prepare such interim analyses as the DMC requests in advance, and the results of these analyses will not be reported to the rest of the trial team. The DMC will then review and make a report to the trial steering committee (TSC) who will advise the trial management group to continue, stop, or they may offer other advice as required. These committee reports will be used by the trial management group to determine if the study should continue or be prematurely terminated, for example, due to extreme success or futility. The DMC and TSC are expected to meet at least twice a year to monitor for quality, futility, and cases of overwhelming success (see SPIRIT item 21a), with their first meeting being at the end of the pre-pilot phase.

### Methods for additional analyses (e.g. subgroup analyses) {20b}

Analyses will be conducted on an intention-to-treat basis with a complier average causal effect analysis (CACE) investigating the causal effect of the intervention on the people who received it as intended by the original group allocation [74]. Subgroup analyses may be conducted, power permitting, to examine possible interactions between baseline anxiety, depression, and severity.

We will examine two levels of adherence: minimal and full. Minimal adherence with the intervention will be defined as the participant attending 2 weeks of the 6-week placements. Full adherence will be defined as the participant attending the full 6-week placement [75].

We will secondarily model return to work using a single day of work as the outcome by way of sensitivity analysis to compare our results with what has become common practice in the literature. Our view is that only a single day of work is not very relevant, although examining this as a secondary analysis will facilitate cross-trial comparisons. We will also model the time to return to work using survival analysis approaches (for example, zero-inflated Poisson regression or a negative binomial model, as best fits our data.)

### Harms

The frequency and percentage (%) of serious adverse events and adverse events in the trial will be compared between the two treatments using the chi-squared test provided the expected values in the cross-tabulation are greater than 5; otherwise, Fisher’s exact test will be used. Odds ratios and 95% confidence intervals will be reported. Adjusted analyses will not be performed for any harm data. The event type, severity assessment, expectedness, and relatedness to intervention will also be summarised by offer of treatment or cohort only.

### Methods in analysis to handle protocol non-adherence and any statistical methods to handle missing data {20c}

The levels and patterns of non-responders at each follow-up time point will be monitored regularly. The levels and patterns of missingness in the primary outcome will first be assessed with the aim of determining the type of missingness (e.g. missing at random, not missing at random). If appropriate and required, as an additional sensitivity analysis, imputation techniques will be used to impute data and estimate the treatment effect.

### Plans to give access to the full protocol, participant-level data, and statistical code {31c}

Anonymised trial data and statistical analysis code may be accessed by contacting the corresponding author upon request. Deidentified data will be stored in the Norwegian Centre for Research Data repository to facilitate this.

### Oversight and monitoring

#### Composition of the coordinating centre and trial steering committee {5d}

The coordinating centre is located in Oslo at Kristiania University College, which is the trial sponsor. Research will be conducted within appropriate legal and ethical frameworks, supported by standard operating procedures and data management policies. The trial working group will meet weekly and consists of the chief investigator and three key members of the trial on-site at Kristiania University College. The trial management group includes all the collaborators and will regularly to oversee trial progress. The TSC will comprise seven members: the chief investigator, trial manager, four independent members recognised as experts in the field of vocational rehabilitation, and an additional user representative. The TSC charter may be requested by contacting the chief investigator. TSC meetings will follow DMC meetings and take place biannually to monitor study progress and provide public and/or professional advice, including review of project reports, protocols, amendments, and adherence to protocol. A user group will comprise six members, two of whom will join the trial management group. The composition of the user group will be a mix of people from the target population. The user group will provide advice from a user perspective and meet quarterly or depending on the need.

#### Composition of the data monitoring committee, its role, and reporting structure {21a}

DMC will comprise three independent experts, one of whom will be an expert statistician and the trial statistician. Only the trial statistician will attend from the trial management group, although the chief investigator may attend an open session if requested by the DMC. The trial statistician will present a report and summary of the analyses for the DMC as these are required by the DMC. The DMC will meet biannually prior to TSC meetings and report to the TSC. DMC will monitor for quality, futility, and extreme success. The DMC charter may be requested by contacting the chief investigator.

### Adverse event reporting and harms {22}

Adverse events will be reported via case managers. Minor adverse events (e.g. a participant being distressed during a session) are logged by the case manager and reported to the trial manager. Serious adverse events related to the study or within the study setting (e.g. during a meeting between the participant and case managers) are reported immediately to the chief investigator in accordance with good clinical practice guidelines. If necessary, the adverse event is reported to the sponsor and to the Norwegian Board of Health Supervision.

### Frequency and plans for auditing trial conduct {23}

If the investigators, sponsor, DMC, or TSC become concerned about any aspects of trial conduct, the trial management group or sponsor will evaluate the need to bring in an independent auditor to resolve trial conduct issues.

### Plans for communicating important protocol amendments to relevant parties (e.g. trial participants, ethical committees) {25}

Protocol amendments will be submitted for the Norwegian Centre for Research Data and Regional Ethics Committee approval if required and revised protocols provided to the funder. Substantial modifications will be communicated to all parties by the chief investigator. Minor administrative modifications that will not affect the conduct of the study will be approved in monthly meetings of the trial management group to consider any further action (e.g. notice to the funder, updating ISRCTN registry, and public announcement if necessary).

### Dissemination plans {31a}

We aim to publish the trial report in a high-impact peer-reviewed journal. We will include user representatives as authors to ensure lay summaries and abstracts are accessible and relevant. We will present the results at national and international conferences on pain, work, and health. We will create a lay report for the consumption of participants and all potential users. We will engage users with persistent pain via a bespoke website and dedicated social media channels, which will be regularly updated to promote resource updates, debates, and relevant news articles. We will create a Norwegian-language manual documenting process, fidelity, and results and make this available in addition to an executive summary. Led by a work and health policy expert, we will develop policy briefs specifically for the consumption of policymakers, so findings can be readily used to inform policymaking.

## Discussion

The project addresses the needs of a large vulnerable unemployed group with persistent pain. A deeper understanding of large diagnosis groups comprising musculoskeletal disorders (which accounts for most persistent pain) may make it possible to give such individuals a longer, healthier, and more rewarding working life. Many with persistent pain may appear to have individually complex obstacles to working. However, a novel multidisciplinary approach, featuring matched work placements and case management (tailored for people with pain), may offer a simple, practical, and effective solution. With international collaborative partners, and experts in chronic pain and work and health, the team has previously developed and established the feasibility of this novel intervention in the UK. Strong user involvement has been integrated since the point of design and will continue throughout trial management, dissemination, and all our comprehensive planned impact activities. This will help to ensure that the intervention, trial, and dissemination of results will be relevant, have a meaningful impact, and help to create new pathways to benefit users and the wider society in the case of cost-effectiveness. Known challenges associated with the trials of interventions such as this include participant recruitment, placement provider recruitment, and timing of intervention. In the English feasibility study, the mean time between baseline measurement and the start of work placement was 94.8 days (SD = 35.5). Additionally, placement providers expressed concern over the time and resources needed to onboard a person into a work placement that is part-time for a limited period (e.g. general preparation, introduction to IT systems, data protection training). We aim to address these concerns by using a broad recruitment strategy informed by our feasibility study and Norwegian trials with similar populations and flexibility over adding additional placement providers [13, 76]. Allowing placement providers to provide training and onboarding prior to the 6-week placement may improve the recruitment of placement providers [13]. A defined timeline from baseline measurement to placement is monitored by the trial manager which might aid the timing of the intervention.

### Implications

There are clear pathways for multiple impact vectors from this work. Our study will provide valuable information on the effectiveness and cost-effectiveness of a biopsychosocial intervention that empowers participants to identify their own obstacles to work and provides a safe environment to overcome those obstacles with support. A cost-effective intervention would have several direct societal impacts. Importantly, it will provide a pathway back to work for an experienced group at a time when the percentage of older people outside the workforce has increased and is unsustainable. This will lead to more years of work and more opportunities and create value for the industry. Working longer will not only decrease the period at the end of working life, reducing demand on welfare services, but create synergy through simultaneously improving health and lowering demand on health services. Family and partners often have care duties for those with persistent pain and relationship strains impact quality of life [60, 77]. Improving work participation would thus improve the quality of life not only for people with pain, but also for partners and families through reducing these strains. Participants in our feasibility study commented that the intervention helped them feel more connected and less alone [13]. Thus, the intervention may not only reduce the national social burden of disability, but also facilitate integration and good society.

## Trial status

Protocol version 1.0 (31/01/2023).

Recruitment start date: January 2023 (with pre-pilot recruitment started in September 2022).

Recruitment end date: December 2023.

## Supplementary Information

**Additional file 1.** ReISE_Grant.

**Additional file 2.** Ethics_402918.

**Additional file 3.** DPIA_693603.

## Data Availability

Upon study completion, a de-identified dataset will be archived in a data repository. Following completion of the project, de-identified study data may be accessed upon request to the corresponding author.

## Abbreviations

DMC: Data monitoring committee
GDPR: General Data Protection Regulation
KUC: Kristiania University College
NAV: Norwegian Labour and Welfare Administration
OsloMet: Oslo Metropolitan University
RTW: Return to work
RCT: Randomised controlled trial
TSC: Trial steering committee
TSD: Services for sensitive data
UiO: University of Oslo

## References

[1] Laerum E, Brage S, Ihlebaek C, Natvig B, Aas E (2013). Et muskel- og skjelettregnskap: forekomst og kostnader knyttet til skader, sykdommer og plager i muskel- og skjelettsystemet: MST-rapport.

[2] Elliott AM, Smith BH, Penny KI, Smith WC, Chambers WA (1999). The epidemiology of chronic pain in the community. Lancet.

[3] NAV. Fortsatt stabilt sykefravær i Oslo 2019 [Available from: https://www.nav.no/no/lokalt/oslo/pressemeldinger/fortsatt-lavest-sykefravaer-i-oslo. Accessed May 27 2020.

[4] Waddell G, Burton AK. Is work good for your health and well-being? London (UK): The Stationery Office; 2006.

[5] Skogli E, Theie MG, Stokke O, Lind LH. Muskel- og skjelettsykdom i Norge: Rammer flest - koster mest. Vurdering av tiltak for å redusere samfunnskostnadene. Oslo (Norway): Menon Economics 2019.

[6] SSB (2020). Labour force survey.

[7] NAV (2020). Disability benefit - quarterly statistics.

[8] Rustoen T, Wahl AK, Hanestad BR, Lerdal A, Paul S, Miaskowski C (2004). Gender differences in chronic pain–findings from a population-based study of Norwegian adults. Pain Manag Nurs.

[9] UNECE National Report on Ageing 2016 – NORWAY (2016). Follow-up to the Regional Implementation Strategy of the Madrid International Action Plan on Ageing.

[10] Linnemorken LTB, Sveinsdottir V, Knutzen T, Rodevand L, Hernaes KH, Reme SE (2018). Protocol for the Individual Placement and Support (IPS) in pain trial: a randomized controlled trial investigating the effectiveness of IPS for patients with chronic pain. BMC Musculoskelet Disord.

[11] Sveinsdottir V, Tveito TH, Bond GR, Grasdal AL, Lie SA, Reme SE (2016). Protocol for the SEED-trial: supported employment and preventing early disability. BMC Public Health.

[12] Waddell G, Burton AK, Kendall N. Vocational rehabilitation – what works, for whom, and when? London (UK): The Stationery Office; 2013.

[13] Froud R, Grant M, Burton K, Foss J, Ellard DR, Seers K (2020). Development and feasibility of an intervention featuring individual supported work placements to aid return to work for unemployed people living with chronic pain. Pilot Feasibility Stud.

[14] Hooper R, Froud RJ, Bremner SA, Perera R, Eldridge S (2013). Cascade diagrams for depicting complex interventions in randomised trials. BMJ..

[15] Kwakkenbos L, Imran M, McCall SJ, McCord KA, Fröbert O, Hemkens LG (2021). CONSORT extension for the reporting of randomised controlled trials conducted using cohorts and routinely collected data (CONSORT-ROUTINE): checklist with explanation and elaboration. BMJ.

[16] Chan AW, Tetzlaff JM, Altman DG, Dickersin K, Moher D (2013). SPIRIT 2013: new guidance for content of clinical trial protocols. Lancet.

[17] Dawson L, Zarin DA, Emanuel EJ, Friedman LM, Chaudhari B, Goodman SN (2009). Considering usual medical care in clinical trial design. PLoS Med..

[18] Kendall N, Burton AK, Main C, Watson P. Tackling musculoskeletal problems - a guide for the clinic and workplace. Identifying obstacles using the psychosocial flags framework. London (UK): The Stationary Office; 2009.

[19] Burton K, Bartys S (2022). The smart return to work plan: part 1: the concepts. Occupational Health at Work..

[20] Etuknwa A, Bartys S, Burton K. The smart return to work plan - part 2: the build. Occupational Health at Work. 2023;19(5):16–25.

[21] Burton AK, Kendall N, McCluskey S, Dibben P. Telephonic support to facilitate return to work: what works, how, and when? Department for Work and Pensions, London (UK); 2013.

[22] Burton AK, Waddell G. Advising patients about work: an evidence-based approach for general practitioners and other healthcare professionals. London (UK): The Stationary Office; 2008.

[23] Burton AK, Waddell G (2007). Health and work - employee’s booklet: the Stationary Office.

[24] Waddell G, Burton AK. Work and health: changing how we think about common health problems. London (UK): The Stationary Office; 2007.

[25] Oiestad BE, Aanesen F, Lochting I, Storheim K, Tingulstad A, Rysstad TL (2020). Study protocol for a randomized controlled trial of the effectiveness of adding motivational interviewing or stratified vocational advice intervention to usual case management on return to work for people with musculoskeletal disorders. The MI-NAV study BMC Musculoskelet Disord.

[26] EU General Data Protection Regulation (GDPR): Regulation (EU) 2016/679 of the European Parliament and of the Council of 27 April 2016 on the protection of natural persons with regard to the processing of personal data and on the free movement of such data, and repealing Directive 95/46/EC (General Data Protection Regulation), OJ 2016 L 119/1. 2016 [Available from: https://gdpr-info.eu/art-6-gdpr/. Accessed June 12 2022

[27] Steckler A, Linnan L (2002). Process evaluation for public health interventions and research.

[28] Bejerholm U, Areberg C, Hofgren C, Sandlund M, Rinaldi M (2015). Individual placement and support in Sweden - a randomized controlled trial. Nord J Psychiatry.

[29] Jensen C, Jensen OK, Nielsen CV (2012). Sustainability of return to work in sick-listed employees with low-back pain. Two-year follow-up in a randomized clinical trial comparing multidisciplinary and brief intervention. BMC Musculoskelet Disord..

[30] McKee-Ryan F, Song Z, Wanberg CR, Kinicki AJ (2005). Psychological and physical well-being during unemployment: a meta-analytic study. J Appl Psychol.

[31] Rueda S, Chambers L, Wilson M, Mustard C, Rourke SB, Bayoumi A (2012). Association of returning to work with better health in working-aged adults: a systematic review. Am J Public Health.

[32] van der Noordt M, H IJ, Droomers M, Proper KI (2014). Health effects of employment: a systematic review of prospective studies. Occup Environ Med..

[33] Norström F, Virtanen P, Hammarström A, Gustafsson PE, Janlert U (2014). How does unemployment affect self-assessed health? A systematic review focusing on subgroup effects. BMC Public Health.

[34] Tennant R, Hiller L, Fishwick R, Platt S, Joseph S, Weich S (2007). The Warwick-Edinburgh Mental Well-being Scale (WEMWBS): development and UK validation. Health Qual Life Outcomes.

[35] Cella D, Choi SW, Condon DM, Schalet B, Hays RD, Rothrock NE (2019). PROMIS(®) adult health profiles: efficient short-form measures of seven health domains. Value Health.

[36] Abma FI, Bültmann U, Amick Iii BC, Arends I, Dorland HF, Flach PA (2018). The Work Role Functioning Questionnaire v2.0 showed consistent factor structure across six working samples. Journal of Occupational Rehabilitation..

[37] Hansen TM, Helland Y, Augestad LA, Rand K, Stavem K, Garratt A (2020). Elicitation of Norwegian EQ-5D-5L values for hypothetical and experience-based health states based on the EuroQol Valuation Technology (EQ-VT) protocol. BMJ Open.

[38] Herdman M, Gudex C, Lloyd A, Janssen M, Kind P, Parkin D (2011). Development and preliminary testing of the new five-level version of EQ-5D (EQ-5D-5L). Qual Life Res.

[39] Smith ORF, Alves DE, Knapstad M, Haug E, Aaro LE (2017). Measuring mental well-being in Norway: validation of the Warwick-Edinburgh Mental Well-being Scale (WEMWBS). BMC Psychiatry.

[40] Garratt AM, Coste J, Rouquette A, Valderas JM (2021). The Norwegian PROMIS-29: psychometric validation in the general population for Norway. Journal of Patient-Reported Outcomes.

[41] Cella D, Choi SW, Condon DM, Schalet B, Hays RD, Rothrock NE (2019). PROMIS® adult health profiles: efficient short-form measures of seven health domains. Value in Health.

[42] Adel M, Akbar R, Ehsan G (2019). Validity and reliability of work ability index (WAI) questionnaire among Iranian workers; a study in petrochemical and car manufacturing industries. J Occup Health.

[43] El Fassi M, Bocquet V, Majery N, Lair ML, Couffignal S, Mairiaux P (2013). Work ability assessment in a worker population: comparison and determinants of Work Ability Index and Work Ability score. BMC Public Health.

[44] Solberg TK, Olsen J-A, Ingebrigtsen T, Hofoss D, Nygaard ØP (2005). Health-related quality of life assessment by the EuroQol-5D can provide cost-utility data in the field of low-back surgery. Eur Spine J.

[45] Garratt AM, Hansen TM, Augestad LA, Rand K, Stavem K (2022). Norwegian population norms for the EQ-5D-5L: results from a general population survey. Qual Life Res.

[46] Garratt AM, Furunes H, Hellum C, Solberg T, Brox JI, Storheim K (2021). Evaluation of the EQ-5D-3L and 5L versions in low back pain patients. Health Qual Life Outcomes.

[47] Garratt AM, Hansen TM, Augestad LA, Rand K, Stavem K (2022). Norwegian population norms for the EQ-5D-5L: results from a general population survey. Qual Life Res.

[48] Smith ORF, Alves DE, Knapstad M, Haug E, Aarø LE (2017). Measuring mental well-being in Norway: validation of the Warwick-Edinburgh Mental Well-being Scale (WEMWBS). BMC Psychiatry.

[49] Garratt AM, Coste J, Rouquette A, Valderas JM (2021). The Norwegian PROMIS-29: psychometric validation in the general population for Norway. J Patient Rep Outcomes.

[50] Oellingrath IM, De Bortoli MM, Svendsen MV, Fell AKM (2019). Lifestyle and work ability in a general working population in Norway: a cross-sectional study. BMJ Open.

[51] Johansen T, Lund T, Jensen C, Momsen AH, Eftedal M, Oyeflaten I (2018). Cross-cultural adaptation of the Work Role Functioning Questionnaire 2.0 to Norwegian and Danish. Work..

[52] Amick BC, Lerner D, Rogers WH, Rooney T, Katz JN (2000). A review of health-related work outcome measures and their uses, and recommended measures. Spine (Phila Pa 1976)..

[53] Aasdahl L, Pape K, Vasseljen O, Johnsen R, Fimland MS (2019). Improved expectations about length of sick leave during occupational rehabilitation is associated with increased work participation. J Occup Rehabil.

[54] Brouwer S, Amick BC, Lee H, Franche R-L, Hogg-Johnson S (2015). The predictive validity of the return-to-work self-efficacy scale for return-to-work outcomes in claimants with musculoskeletal disorders. J Occup Rehabil.

[55] Nøttingnes C, Fersum KV, Reme SE, Moe-Nilssen R, Morken T. Job-related self-efficacy in musculoskeletal disorders - a questionnaire. Tidsskr Nor Laegeforen. 2019. 10.4045/tidsskr.18.0571. 10.4045/tidsskr.18.0571 31429250

[56] Beaton DE, Bombardier C, Guillemin F, Ferraz MB (2000). Guidelines for the process of cross-cultural adaptation of self-report measures. Spine (Phila Pa 1976)..

[57] Johansen T, Lund T, Jensen C, Momsen AH, Eftedal M, Øyeflaten I (2018). Cross-cultural adaptation of the Work Role Functioning Questionnaire 2.0 to Norwegian and Danish. Work..

[58] Chan AW, Tetzlaff JM, Gøtzsche PC, Altman DG, Mann H, Berlin JA (2013). SPIRIT 2013 explanation and elaboration: guidance for protocols of clinical trials. BMJ.

[59] Eldridge SM, Ashby D, Kerry S (2006). Sample size for cluster randomized trials: effect of coefficient of variation of cluster size and analysis method. Int J Epidemiol.

[60] Froud R, Patterson S, Eldridge S, Seale C, Pincus T, Rajendran D (2014). A systematic review and meta-synthesis of the impact of low back pain on people’s lives. BMC Musculoskelet Disord.

[61] Froud R, Underwood M, Carnes D, Eldridge S (2012). Clinicians’ perceptions of reporting methods for back pain trials: a qualitative study. Br J Gen Pract.

[62] Pocock SJ, Simon R (1975). Sequential treatment assignment with balancing for prognostic factors in the controlled clinical trial. Biometrics.

[63] Ryan P. RCT_MINIM: Stata module to assign treatments to subjects in a controlled trial: Boston College Department of Economics; [Available from: https://sociorepec.org/publication.xml?h=repec:boc:bocode:s457029&l=en. Accessed 28 Sep 2022

[64] Altman DG, Bland JM (2005). Treatment allocation by minimisation. BMJ..

[65] Saghaei M (2011). An overview of randomization and minimization programs for randomized clinical trials. J Med Signals Sens.

[66] Saghaei M, Saghaei S (2011). Implementation of an open-source customizable minimization program for allocation of patients to parallel groups in clinical trials. J Biomed Sci Eng.

[67] Morgan AJ, Rapee RM, Bayer JK (2017). Increasing response rates to follow-up questionnaires in health intervention research: randomized controlled trial of a gift card prize incentive. Clin Trials.

[68] Triplet JJ, Momoh E, Kurowicki J, Villarroel LD, Law Ty, Levy JC (2017). E-mail reminders improve completion rates of patient-reported outcome measures. JSES Open Access..

[69] Fox CM, Boardley KLRD (1998). Cost-effectiveness of follow-up strategies in improving the response rate of mail surveys. Ind Mark Manage.

[70] NICE guide to the methods of technology appraisal. London (UK): National Institute for Health & Clinical Excellence (NICE); 2008. 27905712

[71] Dolan P (1997). Modeling valuations for EuroQol health states. Med Care.

[72] Brazier JE, Roberts J (2004). The estimation of a preference-based measure of health from the SF-12. Med Care.

[73] Ritchie J, Lewis J (2003). Qualitative research practice: Sage Publications.

[74] Hewitt CE, Torgerson DJ, Miles JN (2006). Is there another way to take account of noncompliance in randomized controlled trials?. CMAJ.

[75] Hewitt C, Torgerson D, Miles J (2006). Is there another way to take account of noncompliance in randomized controlled trials?. CMAJ.

[76] Sveinsdottir V, Jacobsen HB, Ljosaa TM, Linnemørken LTB, Knutzen T, Ghiasvand R (2022). The individual placement and support (IPS) in Pain trial: a randomized controlled trial of IPS for patients with chronic pain conditions. Pain Med.

[77] Grant M, O’Beirne-Elliman J, Froud R, Underwood M, Seers K (2019). The work of return to work. Challenges of returning to work when you have chronic pain: a meta-ethnography. BMJ Open..

