# Supplementary material for: Individual supported work placements (ReISE) for improving sustained return to work in unemployed people with persistent pain: study protocol for a cohort randomised controlled trial with embedded economic and process evaluations
Source: Trials. 2023 Mar 11;24:179. doi: 10.1186/s13063-023-07211-5 (PMC10006572; doi:10.1186/s13063-023-07211-5)
Supplement: Supplementary file 1 — Additional file 1. ReISE_Grant. [file 13063_2023_7211_MOESM1_ESM.pdf]

The Research Council of Norway

HØYSKOLEN KRISTIANIA - ERNST G MORTENSENS STIFTELSE
Postboks 1190 Sentrum
0107 OSLO
Norge

**Enquiries to**	**Our ref.:**	**Oslo,**
Siri H. Hollekim Haaland	326732/ABHO	02.07.2021
+4722037513	**Application received:**	
shh@forskningsradet.no	17.02.2021	

Call for proposals:	Samarbeidsprosjekt for å møte utfordringer i samfunn og næringsliv, 17.02.2021
Project:	Returning people with persistent pain to work using Individual Supported Employment placements (ReISE)
Project No.	326732
Project Owner:	HØYSKOLEN KRISTIANIA - ERNST G MORTENSENS STIFTELSE
Project Manager:	Robert James Froud
Project Administrator:	Trine Johansen Meza

Dear Sir/Madam,

We are pleased to inform you that the Research Council of Norway has made a conditional decision to grant up to 11 842 000 for your project, contingent upon the revision of the grant application as stipulated below. The deadline for submission of the revised grant application and project description is 31.08.2021. Before you revise the grant application, the project is obligated to have a contract meeting with the representative case officer for this project to clarify closer important aspects to be revised before a final project acceptance.

A list of the projects awarded funding along with a list of the referees used to assess the grant applications under this call for proposals may be found on the Research Council's website. Please contact the relevant case officer for more information about which experts reviewed your grant application.

Revising the grant application

You need to update or confirm the objectives, progress plan, and items relating to the budget, project description and other relevant components of the grant application.

To revise the grant application, go to “Applications in eSøknad” on My RCN web. The application is now available as “Revised”. You will need to provide additional budget information in the revised grant application, so the application form may look somewhat different from the original application you submitted. Please note that you must click on *Go to submit* in order to send us the updated grant application.

The content of the revised grant application will be used as the basis for the contract between the Research Council and the Project Owner. Read more about the contracts for R&D projects here.

We recommend that you begin the project revision immediately. Feel free to contact the case officer if you have any questions.

Stipulations:

- The mentioned grant amount is an upper limit and can be adjusted after reviewing the project's cost and financing plan, ensuring that the allocation of funds is in line with the state aid rules.
- When awarding state aid to collaborating partners, a declaration form for each of the partners must be submitted together with the revised grant application.
- Only in special occasions the project partners can be changed. If changes are needed, this must be approved by the Research Council and communicated to existing and new partners before entering into the contract.
- The project must start between 1. July 2021 and 1. December 2021.

The progress plan:
a. We cannot see that the activities listed qualify as fundamental research and we ask that you re-evaluate these categories. Please find more information here (especially under "Important definitions for article 25"): https://www.forskningsradet.no/en/apply-for-funding/funding-from-the-research-council/Conditions-for-awarding-state-aid/
b. We ask that you split up your main activities into more detail. As of now, the progress plan does not give a lot of detail on activities and milestones. Please note that changes here also entails updates to the first table under Budget.
Budget
a. Please provide more details, under Specification or in separate document, on how you have calculated the personal costs, specifically hourly rates and man months per participant. Please make sure you are using the Research Council's guidelines: https://www.forskningsradet.no/en/apply-for-funding/Budget/
b. You list several costs under Specification that needs more details. Specifically, please specify whether costs like travel and phones are costs occurring due to the research, or if this is a normal part of project participants' employment. If the latter, these costs are included as

overhead in hourly rates.
c. You have included large sums for travel and accommodation – are sums calculated based on assessments of travel versus digital meetings,? If so, please confirm.
d. Rådet for muskelskjeletthelse is considered "Other sector" and costs must be moved accordingly in Cost code table.

We are not asking you to amend the project description, but if you do, please highlight areas where you have edited text.

Provide a popular science presentation
You must write a popular science presentation in both Norwegian and English in your revised grant application. It is important to write both the project summary and the popular science presentation in a way that is understandable to individuals who do not have the relevant scientific background. Make sure you do not include any confidential information from the project in these texts. Please note that information about the project will be published in the Research Council's project databank once the revised grant application has been approved. Read more about the Project Databank and how to write good popular science presentations here.

Required submission of data management plans
In connection with the revision of the grant application, R&D-performing organisations or companies must assess the need to develop a data management plan for all projects that have been granted research funding. These plans are normally required if the project collects or in some other way produces research data. If the Project Owner decides that the project does not need to develop a data management plan, an explanation of this must be provided in the grant application. Information about the archive solution(s) to be used for storing the data is to be provided in the data management plan. Click here for more information about data management plans.

Collaboration agreements
The Project Owner is responsible for ensuring that written agreements are signed with all the relevant partners taking part in the project. We encourage all recipients of grant allocations to **start the process of drawing up collaboration agreements immediately**. Suggestions regarding elements to include in an agreement may be found here.

A copy of all signed collaboration agreements is to be attached to the contract when this is returned to the Research Council. You must return the contract documents within three months after we have made it available for you on My RCN Web. The contract will not enter into force until the collaboration agreements are in place. Read more about the "General Terms and Conditions for R&D Projects" here.

Information about scientific assessment and allocation decisions
We have enclosed an overview of the review of your proposal with the general mark for your grant application and the marks assigned to each individual criterion. We have also attached

Norges forskningsråd/
The Research Council of Norway
Drammensveien 288
Postboks 564
NO–1327 Lysaker

Telefon +47 22 03 70 00
Telefaks +47 22 03 70 01
post@forskningsradet.no
www.forskningsradet.no
Org.nr. 970141669

All post og e-post som inngår i saksbehandlingen, bes adressert til Norges forskningsråd og ikke til enkeltpersoner.

Kindly address all mail and e-mail to the Research Council of Norway, not to individual staff.

the assessments of the panel of referees. These assessments are being forwarded to provide you with scientific feedback, and do not comprise the grounds for the funding decision. Please notice that applicants are not permitted to contact the referees.

Limited right to lodge a complaint

The decision of the Research Council is exempt from provisions of the Norwegian Public Administration Act regarding complaints to a superior agency. However, the Research Council does allow complaints within a restricted framework. For more information, see the Research Council's website. Please note that it is *not* permitted to submit complaints relating to the academic or expert assessments and priorities that form the basis for the decision to reject the application.

The deadline for submitting a complaint is 27. August 2021. Complaints must be submitted in writing and may only be put forth by the Project Owner (institution) via the designated project administrator.

Insider information

If you become aware that information provided to the Research Council comprises insider information in accordance with the Securities Trading Act, we ask you please to inform us of this.

Do you have questions?

Please feel free to contact the case officer Siri H. Hollekim Haaland by email shh@forskningsradet.no if you have any questions. Please be sure to include the reference number 326732 on all enquiries to the Research Council relating to this project.

Yours sincerely,

The Research Council of Norway

Vidar Sørhus
Director

Siri H. Hollekim Haaland
Case Officer

Approved and expedited electronically without signature

Attachments

Norges forskningsråd/
The Research Council of Norway
Drammensveien 288
Postboks 564
NO–1327 Lysaker

Telefon +47 22 03 70 00
Telefaks +47 22 03 70 01
post@forskningsradet.no
www.forskningsradet.no
Org.nr. 970141669

All post og e-post som inngår i saksbehandlingen, bes adressert til Norges forskningsråd og ikke til enkeltpersoner.

Kindly address all mail and e-mail to the Research Council of Norway, not to individual staff.
