# Supplementary material for: Individual supported work placements (ReISE) for improving sustained return to work in unemployed people with persistent pain: study protocol for a cohort randomised controlled trial with embedded economic and process evaluations
Source: Trials. 2023 Mar 11;24:179. doi: 10.1186/s13063-023-07211-5 (PMC10006572; doi:10.1186/s13063-023-07211-5)
Supplement: Supplementary file 2 — Additional file 2. Ethics_402918. [file 13063_2023_7211_MOESM2_ESM.pdf]

REK
REGIONALE KOMITEER FOR MEDISINSK OG HELSEFAGLIG FORSKNINGSETIKK

Regional Committee for Medical & Health Research Ethics
South East Norway, Section A
Postbox 1130 Blindern
NO-0318 Oslo
Norway

Phone: + 47 22 84 5512
E-mail: a.s.kavli@medisin.uio.no
Webportal: http://helseforskning.etikkom.no

Our ref.: 402918
IRB ref: IRB00001871

Date: 1th of February 2023

To whom it may concern,

Re: REC Letter of Confirmation

I am writing in reference to a request from Pål Andre Thorsø Amundsen via e-mail dated 1st of February 2023, regarding a Letter of Confirmation in English.

Confirmation
We hereby confirm that Regional Committee for Medical & Health Research Ethics, Section A, South East Norway, approved the Research Project "*Returning people with persistent pain to work using Individual Supported Employment placements (ReISE)*" (Norwegian title: "Arbeidsplassering med individuelle støttetiltak for å hjelpe personer med langvarige smerter tilbake i arbeid (ReISE)") on the 16th of May 2022. The Project Manager for the study is Robert Froud and the Institution Responsible for Research is Kristiania University College.

The approval has been given on the basis that Research Project will be implemented as described in the Research Protocol.

Ethics Committee System
The Ethics Committee System in Norway consists of seven Independent Regional Committees with authority to either approve or disapprove Medical Research Studies conducted within Norway, or by Norwegian Institutions, in accordance with the Act on Medical and Health Research (2008).

Please do not hesitate to contact the Regional Committee for Medical and Health Research Ethics Section South East A (REK Sør-Øst A) if further information is required, as we are happy to be of assistance.

Yours faithfully,

Kristian Bjøro
Chair of the Regional Committee for Medical
& Health Research Ethics of South East Norway,
Section A

Anne Schiøtz Kavli
Senior Executive Officer
