# Supplementary material for: Individual supported work placements (ReISE) for improving sustained return to work in unemployed people with persistent pain: study protocol for a cohort randomised controlled trial with embedded economic and process evaluations
Source: Trials. 2023 Mar 11;24:179. doi: 10.1186/s13063-023-07211-5 (PMC10006572; doi:10.1186/s13063-023-07211-5)
Supplement: Supplementary file 3 — Additional file 3. DPIA_693603. [file 13063_2023_7211_MOESM3_ESM.pdf]

OSLOMET

Til: The Norwegian Agency for Shared Services in Education and Research (Sikt)

Dato: 21. May 2022

## Approval of Data protection impact assessment (DPIA)

The Head of Department of Physiotherapy at Faculty of Health Sciences at OsloMet, Hege Bentzen, approves the Data protection impact assessment conducted by Kristiania University College, OsloMet and Manpower AS for «Returning people with persistent pain to work using Individual Supported Employment placements (ReISE)» with Sikt project number «693603». The project meets the requirements of the privacy legislation, and can start as described in the notification form. In the event of changes, Sikt will be contacted and my help with a new assessment.

Regards

*Hege Bentzen* ______________________ Ingrid S. Jacobsen

Hege Bentzen
The Head of Department, Physio, HV, OsloMet

Ingrid S. Jacobsen
The Data Protection Officer, OsloMet
